# Interaction of Dietary Sodium-to-potassium Ratio and Dinner Energy Ratio on Prevalence of Hypertension in Inner Mongolia, China

**DOI:** 10.2188/jea.JE20220045

**Published:** 2023-11-05

**Authors:** Huiqiu Zheng, Yanling Wang, Bo Yang, Jing Wu, Yonggang Qian, Wenrui Wang, Xuemei Wang

**Affiliations:** 1 Center for Data Science in Health and Medicine, School of Public Health, Inner Mongolia Medical University, Hohhot, China; 2 School of Public Health and Management, Wenzhou Medical University, Wenzhou, China; 3 National Institute for Chronic and Non-Communicable Disease Control and Prevention, Chinese Center for Disease Control and Prevention, Beijing, China; 4 Department of Chronic Disease Control and Prevention, Inner Mongolia Center for Disease Control and Prevention, Hohhot, China

**Keywords:** hypertension, dinner energy ratio, dietary sodium-to-potassium ratio, interaction

## Abstract

**Background:**

Hypertension is one of the most common chronic diseases, and dietary factors play an important role in hypertension. We examined the interaction of dietary sodium-to-potassium (Na/K) ratio and dinner energy ratio on hypertension.

**Methods:**

We conducted this study using data from the cross-sectional National Survey for Nutrition and Adult Chronic Disease in 2015 in Inner Mongolia, China. Dietary data were collected using 24-hour diet records with food weights across 3 consecutive days. Logistic regression was used to determine the interaction of dinner energy ratio and dietary Na/K ratio on hypertension.

**Results:**

A total of 1,861 participants were included in this study, and 914 individuals were hypertensive (49.1%). Dinner energy ratio and high dietary Na/K ratio were independently related to high prevalence of hypertension. A formal test showed that dinner energy ratio interacted significantly with dietary Na/K ratio on hypertension (*P* < 0.001), with an adjusted odds ratio (OR) of 1.119 (95% confidence interval [CI], 1.040–1.203). Participants whose dinner energy ratio greater than 39.1% and dietary Na/K ratio of 3.625–6.053 had the highest OR of hypertension prevalence, with an adjusted OR of 2.984 (95% CI, 1.758–5.066), compared with participants with dinner energy ratio of 30.2–39.1%, and dietary Na/K ratio less than 2.348.

**Conclusion:**

Our study highlighted the interactive effect of dinner energy ratio and dietary Na/K ratio on hypertension among adults in Inner Mongolia. We advocated a balanced diet (dinner energy ratio not small or large) and a low dietary Na/K ratio for reducing the prevalence of hypertension.

## INTRODUCTION

Hypertension is one of the most common chronic diseases and a principal risk factor for death and disability worldwide.^1^^,^^2^ Two-thirds of the world’s patients with hypertension are in economically developing countries.^3^ China is the largest economically developing country in the world. In the past 30 years, the prevalence rate of hypertension was on the rise in China, and about one-third of the adults suffered from hypertension.^4^

It is well known that dietary factors play an important role in hypertension,^5^ and about half of hypertension cases are related to an unhealthy lifestyle. Likewise, high sodium intake is a risk factor for hypertension.^6^ However, the blood-pressure-lowering effects of potassium intake are greater than the blood-pressure-raising effects of high sodium intake.^7^ Recent studies have shown that the dietary sodium-to-potassium (Na/K) ratio has a stronger effect on the risk of hypertension compared with sodium or potassium alone.^8^^–^^10^ A cohort study from Tohoku Medical Megabank Project also showed that dietary Na/K ratio was associated with home hypertension.^11^

Additionally, excessive energy intake is a risk factor for hypertension.^12^ The control of energy intake is the primary method for the prevention and control of hypertension, but few studies have examined the relationship between energy intake of meals and high blood pressure, and their results were inconsistent.^13^^–^^15^ Timing for food has also become an important role of cardiovascular disease risk factors.^16^^,^^17^ In a recent 11-year cohort study of 25,220 participants among middle-aged Chinese adults, a dinner-dominant meal pattern was related to uncontrolled hypertension.^18^

Inner Mongolia has a higher prevalence of hypertension than other regions in China, but the awareness rate and the control rate were low.^19^^,^^20^ Inner Mongolia is located on the northern border of China, and residents have unique dietary and lifestyle habits.^21^ Inner Mongolia has vast grasslands and is relatively developed in animal husbandry, rich in horses, cattle, and sheep.^22^ Inner Mongolians like to eat more meat and dried jerky, which has a strong taste and high sodium content, especially for dinner. However, there are fewer foods rich in potassium, such as seafood, fresh fruit, and vegetables, which is likely to be related to high dietary Na/K ratio.^23^^,^^24^

High dietary Na/K ratio intake and unbalanced dinner energy intake are prevalent in Inner Mongolia.^25^^,^^26^ Both factors play an important role in high blood pressure. A secondary analysis of the Dietary Approaches to Stop Hypertension-Sodium (DASH) trial from America showed that energy intake affected the relationship between sodium intake and high blood pressure.^27^ Moreover, high dietary sodium-to-potassium was a risk factor for hypertension.^28^ However, there is limited evidence regarding the interactive effect of dinner energy ratio and dietary Na/K ratio on hypertension. Therefore, in order to expand the present theoretical basis for hypertension prevention, we explored the association between the effects of dinner energy ratio and dietary Na/K ratio on hypertension among adults in Inner Mongolia. Particularly, we examined whether dinner energy ratio and dietary Na/K ratio have synergistic effects on hypertension and whether have positive or negative interactions.

## METHODS

### Study design

The China National Survey for Nutrition and Adult Chronic Disease, which used a stratified, multi-stage, and random sampling method, was conducted at surveillance sites in 31 provinces, autonomous regions, municipalities directly under the Central Government, and Xinjiang’s Production and Construction Corps in China. The China National Survey for Nutrition and Adult Chronic Disease included dietary and non-dietary surveys of 185,000 participants. Participants was divided into dietary survey and non-dietary survey participants. The participants in this study were only the dietary survey participants in China National Survey for Nutrition and Adult Chronic Disease in Inner Mongolia Autonomous Region, and the replacement rate was less than 35%.^29^ Information was collected, including general demographic characteristics, lifestyle, hypertension prevalence rate, dietary behavioral habits, and daily food intake.

Our study was approved by the Ethical Committee of the National Institute for Nutrition and Food Safety, Chinese Center for Disease Control and Prevention (No. 201519-A). All participants provided written informed consent before the start of the investigation.

### Data collection

We used 24-hour recall record with food weight across 3 consecutive days to collect dietary data using a method previously reported by Wang et al.^30^ The units of dietary sodium and potassium were g/day. We calculate dietary sodium and potassium from the average of the 3 days’ meals and condiments and calculated the ratio of dietary sodium to potassium. For the current inconsistent cut-off level of Na/K ratio and the sample size of the interaction subgroup, we categorized dietary Na/K ratio into four groups according to quartiles (Q), which were as follows for Q1 through Q4: <2.348, 2.348–3.625, 3.625–6.053, and >6.053. Dinner energy ratio was the proportion of dinner energy to the total energy of the whole day. Since 30–40% of the total energy consumed at dinner is considered healthy in China^18^ and also taking into account the sample size of the interaction subgroup, we categorized dinner energy ratio into three groups according to tertiles (T), which were as follows for T1 through T3: <30.2%, 30.2–39.1%, and >39.1%. Blood pressure was measured at the experimental site. The subjects had to rest quietly for 5 minutes before their blood pressure was measured. A total of three blood pressure measurements were taken, with an interval 1 minute between measurements, and the average value was calculated.^31^ Hypertension was defined as average systolic blood pressure ≥140 mm Hg and/or average diastolic blood pressure ≥90 mm Hg or currently taking medicine for hypertension.^32^ The antihypertensive treatment in the definition of hypertension was self-reported.

### Other variables

Age (years) was a quantitative variable. Tumor, cardiovascular disease (myocardial infarction, atrial fibrillation, stenocardia, and stroke), and kidney disease were self-reported and were all categorized as yes or no. On the basis of regional characteristics, ethnic group was categorized as Han, Mongolian, or other ethnic minority (all minorities living in Inner Mongolia except Han and Mongolian). Marital status was categorized as married, unmarried, or another marital status (widowed/divorced/cohabiting/separated). Occupation was categorized as agriculture and farming, production equipment operation and service, enterprises, and public institutions, unemployed, homemaker, retired, or other occupation.

Physical activity was evaluated using the international physical activity questionnaire,^33^ which included a week of various types of physical activity (job-related physical activity; transportation physical activity; housework and caring for family; recreation, sport, and leisure-time physical activity; resting behavior; sleeping behavior). Physical activity was a quantitative variable.

Alcohol consumption was collected using 24-hour recall surveys across 3 consecutive days. Beverage type (liquor with high/low alcohol content, wine, rice wine, yellow rice wine or beer) and amount consumed were measured on 3 consecutive days. Alcohol consumption was calculated as one standard drinking unit equal to 10 grams of alcohol^31^ and categorized as non-excessive drinking (<25 g/day for men and <15 g/day for women) or excessive drinking (≥25 g/day for men and ≥15 g/day for women) according to the 2016 Dietary Guidelines for Chinese Residents.^31^

Smoking was categorized as non-current smoker (never smoked or previously smoked but quit) and current smoker (smoked at least one cigarette/day for more than 1 year and smoking now). Body mass index (BMI; kg/m^2^) was calculated using height and weight, which were measured at the experimental site. BMI was categorized as normal or underweight (≤23.9 kg/m^2^), overweight (24.0–27.9 kg/m^2^), or obese (≥28.0 kg/m^2^), according to the recommended standards issued by a working group on obesity in China.^34^ Because there were few people in the standard underweight category, we combined the normal and underweight categories.

### Statistical analysis

The sample size was estimated according to a stratified random sampling using the formula as follows^35^:

$$n=\text{deff}\frac{u^{2}p(1-p)}{d^{2}}$$

Parameter estimation was based on the hypertension prevalence of the surveillance survey of chronic disease and nutrition in Inner Mongolia in 2013 (*P* = 39.98%). We considered a 0.01 two-sided significance level, a 0.02 permissible δ error value, and a 10% non-response rate, and we estimated the sample size to be 824. In total, 1,861 participants were included, and the statistical power (1 − *β*) was 90.77%. Power Analysis and Sample Size (PASS) software (NCSS) was used to calculate the statistical power.

Quantitative variables were expressed as means and standard deviations and were analyzed using the *t* test. Categorical variables were expressed as numbers and percentages and were analyzed using the χ^2^ test. Those variables with a *P*-value of less than 0.10 in univariate analysis were included in multivariable analysis. Sensitivity analyses were performed by separately excluding patients with tumor, cardiovascular disease, and chronic urinary system disease. Multivariable logistic regression models^36^ were used to examine the interactions of dinner energy ratio and dietary Na/K ratio on hypertension. Complete case analysis was used in this study. *P* < 0.05 was considered statistically significant. IBM SPSS, Version 25.0 was used for all analyses (IBM Corp., Armonk, NY, USA).

## RESULTS

### General characteristics of the study participants

In total, 1,861 (47.8% men and 52.2% women) participants were included in this study, which were all participants from the dietary survey of China National Survey for Nutrition and Adult Chronic Disease in Inner Mongolia adults. The mean age was 53.1 (standard deviation [SD], 12.6) years. Among the participants, 81.8% were Han, and 93.0% were married; 42.9% engaged in agriculture, forestry, animal husbandry, and fishery; 68.5% were non-current smokers; 10.0% were excessive drinkers; 39.2% had a family history of hypertension; 60.5% were overweight or obese, 5.4% were diagnosed with tumors, 9.5% were diagnosed with other cardiovascular diseases, and 9.7% were diagnosed with chronic urinary system disease. The average daily total energy intake was 1,631.8 (SD, 727.2) kcal. The average physical activity level per week was 5,915.0 (SD, 5,118.3) metabolic equivalents of task (METs)-min. The average daily dietary sodium intake was 5.1 (SD, 4.1) g, the average daily dietary potassium intake was 1.2 (SD, 0.7) g, and the average daily dietary Na/K ratio was 5.0 (SD, 4.3). Of the 1,861 respondents, the average dinner energy ratio was 35.0% (SD, 11.2%), and 577 (33.3%) had a dinner energy ratio between 30.2% and 39.1%. eTable 1 shows the characteristics of the subjects in each of the tertile of the dinner energy ratio. People whose dinner energy ratio less than 30.2% (T1) had a higher dietary Na/K ratio, an older age, a lower percentage of married status, and a higher percentage of tumor or cardiovascular disease compared with people whose dinner energy ratio of 30.2–39.1% (T2). Of the 1,861 respondents, 1,395 (75.0%) had a dietary Na/K ratio >2.348 (Table 1). Dinner energy ratio, dietary Na/K ratio, dietary potassium, age, marital status, occupation, family history of hypertension, BMI, physical activity, cardiovascular disease, and chronic urinary system disease were statistically different in participants with and without hypertension (*P* < 0.05) (Table 1).

**Table 1.** General characteristics of study participants between hypertensive and normotensive groups

Variables	Hypertension^b^, *n* (%)	Non-Hypertension, *n* (%)	Total, *n* (%)	*t*/χ^2^	*P*-value
	914 (49.1)	947 (50.9)	1,861 (100.0)		
Age, years, mean (SD)	57.2 (11.5)	49.2 (12.4)	53.1 (12.6)	14.520	<0.001^a^
Sex					
Men	463 (50.7)	426 (45.0)	889 (47.8)	5.998	0.014^a^
Women	451 (49.3)	521 (55.0)	972 (52.2)		
Ethnicity					
Han	745 (81.5)	777 (82.0)	1,522 (81.8)	0.091	0.955
Mongolian	135 (14.8)	136 (14.4)	271 (14.6)		
Other ethnic minority	34 (3.7)	34 (3.6)	68 (3.6)		
Marital status					
Married	840 (92.3)	885 (93.7)	1,725 (93.0)	16.831	<0.001^a^
Unmarried	15 (1.6)	33 (3.5)	48 (2.6)		
Other marital status	55 (6.0)	27 (2.9)	82 (4.4)		
Occupation					
Agriculture and farming	405 (44.3)	394 (41.6)	799 (42.9)	28.284	<0.001^a^
Production equipment operation and service	75 (8.2)	108 (11.4)	183 (9.8)		
Enterprises and public institutions	33 (3.6)	57 (6.0)	90 (4.8)		
Unemployed	31 (3.4)	32 (3.4)	63 (3.4)		
Homemaker	159 (17.4)	163 (17.2)	322 (17.3)		
Retired	98 (10.7)	53 (5.6)	151 (8.1)		
Other occupation	113 (12.4)	140 (14.8)	253 (13.6)		
Family history of hypertension					
Yes	407 (44.5)	322 (34.0)	729 (39.2)	21.633	<0.001^a^
No	507 (55.5)	625 (66.0)	1,132 (60.8)		
Total energy intake, kcal/day, mean (SD)	1,577.5 (690.1)	1,684.2 (757.9)	1,631.8 (727.2)	3.169	0.002^a^
Dinner energy ratio, %, mean (SD)	35.0 (11.3)	35.0 (11.1)	35.0 (11.2)	−0.523	0.601
T1 (<30.2)	294 (35.2)	283 (31.6)	577 (33.3)	10.888	0.004^a^
T2 (30.2–39.1%)	246 (29.5)	331 (36.9)	577 (33.3)		
T3 (>39.1)	295 (35.3)	282 (31.5)	577 (33.3)		
Dietary Na/K ratio, g/g, mean (SD)	5.4 (4.6)	4.7 (4.2)	5.0 (4.3)	−3.714	<0.001
Q1 (<2.348)	203 (22.2)	262 (27.7)	465 (25.0)	17.647	<0.001^a^
Q2 (2.348–3.625)	212 (23.2)	253 (26.7)	465 (25.0)		
Q3 (3.625–6.053)	237 (26.0)	228 (24.1)	465 (25.0)		
Q4 (>6.053)	261 (28.6)	204 (21.5)	465 (25.0)		
Dietary sodium, g, mean (SD)	5.2 (4.2)	4.9 (3.9)	5.1 (4.1)	1.489	0.137
Dietary potassium, g, mean (SD)	1.1 (0.6)	1.3 (0.7)	1.2 (0.7)	5.094	<0.001^a^
Excessive drinking					
No	791 (86.5)	884 (93.3)	1,675 (90.0)	23.941	<0.001^a^
Yes	123 (13.5)	63 (6.7)	186 (10.0)		
Smoking					
Non-current smoker	633 (69.3)	641 (67.7)	1,274 (68.5)	0.530	0.467
Current smoker	281 (30.7)	306 (32.3)	587 (31.5)		
BMI, kg/m^2^					
Underweight/normal ( ⩽ 23.9 kg/m^2^)	278 (30.5)	444 (48.5)	722 (39.5)	78.935	<0.001^a^
Overweight (24.0–27.9 kg/m^2^)	401 (44.1)	356 (38.9)	757 (41.5)		
Obese ( ⩾ 28.0 kg/m^2^)	231 (25.4)	116 (12.7)	347 (19.0)		
Physical activity, mean METs-min/w (SD)	5,582.9 (4,764.5)	6,225.2 (5,412.2)	5,915.0 (5,118.3)	2.575	0.010^a^
Tumor					
No	861 (94.2)	898 (94.9)	1,759 (94.6)	0.475	0.491
Yes	53 (5.8)	48 (5.1)	101 (5.4)		
Cardiovascular disease					
No	786 (86.0)	898 (94.9)	1,684 (90.5)	43.275	<0.001^a^
Yes	128 (14.0)	48 (5.1)	176 (9.5)		
Chronic urinary system disease					
No	801 (87.6)	878 (92.8)	1,679 (90.3)	14.173	<0.001^a^
Yes	113 (12.4)	68 (7.2)	181 (9.7)		

BMI, body mass index; METs-min/w, metabolic equivalents of task-minutes/week; Na/K ratio, sodium-to-potassium ratio; Q, quartile; SD, standard deviation; T, tertile.

^a^*P* < 0.05.

^b^Hypertension was defined as average systolic blood pressure ≥140 mm Hg and/or average diastolic blood pressure ≥90 mm Hg or currently receiving hypertension treatments.

### Associations among dinner energy ratio, dietary Na/K ratio, sodium, potassium and hypertension

Table 2 showed the independent effects of dinner energy ratio and dietary Na/K ratio on the prevalence of hypertension. Participants with dinner energy ratio greater than 39.1% and dietary Na/K ratio greater than 6.053 had the highest odds ratios (ORs) of hypertension (Table 2). After adjustment for confounders, compared with people whose dietary Na/K ratio less than 2.348 (Q1), increasing dietary Na/K ratio was independently related to an increased prevalence of hypertension (Q2 vs Q1: adjusted OR 1.255; 95% confidence interval [CI], 0.919–1.714; Q3 vs Q1: adjusted OR 1.409; 95% CI, 1.027–1.934; Q4 vs Q1: adjusted OR 1.624; 95% CI, 1.174–2.247). After adjustment for confounders, compared with people whose dinner energy ratio was 30.2–39.1% (T2), a high or a low dinner energy ratio were independently related to an increased prevalence of hypertension (T1 vs T2: adjusted OR 1.371; 95% CI, 1.036–1.814; T3 vs T2: adjusted OR 1.716; 95% CI, 1.302–2.261). The intake of dietary sodium and potassium were both related to the prevalence of hypertension, with adjusted ORs of hypertension of 1.030 (95% CI, 1.003–1.058) and 0.697 (95% CI, 0.583–0.832), respectively (eTable 2). Multivariable logistic regression analysis revealed a significant interaction between dinner energy ratio and dietary Na/K ratio on the prevalence of hypertension, with an adjusted OR of 1.119 (95% CI, 1.040–1.203) (Table 2).

**Table 2.** Odds ratios of hypertension for dinner energy ratio and dietary Na/K ratio (*n* = 1,504)

Variables	Hypertension^c^, *n* (%)	χ^2^	*P*-value	Adjusted^a^	

				OR (95% CI)	*P*-value
**Dinner energy ratio**		10.888	0.004		
T1 (<30.2%)	294 (35.2)			1.371 (1.036–1.814)	0.027^b^
T2 (30.2–39.1%)	246 (29.5)			1.000 (reference)	
T3 (>39.1%)	295 (35.3)			1.716 (1.302–2.261)	<0.001^b^
**Dietary Na/K ratio**		17.647	<0.001		
Q1 (<2.348)	203 (22.2)			1.000 (reference)	
Q2 (2.348–3.625)	212 (23.2)			1.255 (0.919–1.714)	0.153
Q3 (3.625–6.053)	237 (26.0)			1.409 (1.027–1.934)	0.034^b^
Q4 (>6.053)	261 (28.6)			1.624 (1.174–2.247)	0.003^b^
**Dinner energy ratio × Dietary Na/K ratio**	—	—	—	1.119 (1.040–1.203)	0.002^b^

CI, confidence interval; Na/K ratio, sodium-to-potassium ratio; OR, odds ratio; Q, quartile; SD, standard deviation; T, tertile.

^a^Adjusted for age, sex, marital status, occupation, body mass index, physical activity, excessive drinking, family history of hypertension, cardiovascular disease, chronic urinary system disease.

^b^*P* < 0.05.

^c^Hypertension was defined as average systolic blood pressure ≥140 mm Hg and/or average diastolic blood pressure ≥90 mm Hg or currently receiving hypertension treatments.

### Interaction of dinner energy ratio and dietary Na/K ratio on hypertension

Finally, 1,504 participants were included in the adjusted multivariable logistic regression model to determine the interaction of dinner energy ratio and dietary Na/K ratio on hypertension (Table 4). Participants were categorized into twelve subgroups considering dinner energy ratio and dietary Na/K ratio jointly. The participants with a dinner energy ratio greater than 39.1% and a dietary Na/K ratio greater than 6.053 had the highest prevalence of hypertension, whereas the participants with a dinner energy ratio of 30.2–39.1% and a dietary Na/K ratio less than 2.348 had the lowest prevalence of hypertension (*P* = 0.002) (Table 3). Multivariable logistic regression analysis showed that significant two-factor interactions occurred between dietary Na/K ratio and dinner energy ratio for high prevalence hypertension by comparing these twelve subgroups (*P* = 0.001) (Table 4). Compared with participants who with a dinner energy ratio of 30.2–39.1% and a dietary Na/K ratio less than 2.348, participants with a dinner energy ratio of 30.2–39.1% and a dietary Na/K ratio greater than 6.053 had a higher prevalence of hypertension (adjusted OR 2.039; 95% CI, 1.152–3.609), participants who with a dinner energy ratio less than 30.2% and a dietary Na/K ratio of 3.625–6.053 had a higher prevalence of hypertension (adjusted OR 2.049; 95% CI, 1.202–3.493), and participants who with a dinner energy ratio greater than 39.1% and a dietary Na/K ratio of 2.348–3.625 had a higher prevalence of hypertension (adjusted OR 2.166; 95% CI, 1.306–3.593) (Table 4).

**Table 3.** Prevalence of hypertension in different population subgroups of dinner energy ratio and dietary Na/K ratio (*n* = 1,731)

Variables	Total, *n*	Hypertension^c^, *n* (%)	χ^2^	*P*-value
**Subgroups**			28.844	0.002
Dinner energy ratio<30.2% (Dietary Na/K ratio<2.348)	156	74 (47.4)		
Dinner energy ratio<30.2% (2.348< Dietary Na/K ratio<3.625)	115	52 (45.2)		
Dinner energy ratio<30.2% (3.625< Dietary Na/K ratio<6.053)	149	80 (53.7)		
Dinner energy ratio<30.2% (Dietary Na/K ratio>6.053)	157	88 (56.1)		
30.2%<Dinner energy ratio<39.1% (Dietary Na/K ratio<2.348)^a^	167	63 (37.7)		
30.2%<Dinner energy ratio<39.1% (2.348< Dietary Na/K ratio<3.625)	160	67 (41.9)		
30.2%<Dinner energy ratio<39.1% (3.625< Dietary Na/K ratio<6.053)	130	55 (42.3)		
30.2%<Dinner energy ratio<39.1% (Dietary Na/K ratio>6.053)	120	61 (50.8)		
Dinner energy ratio>39.1% (Dietary Na/K ratio<2.348)	120	51 (42.5)		
Dinner energy ratio>39.1% (2.348< Dietary Na/K ratio<3.625)	158	74 (46.8)		
Dinner energy ratio>39.1% (3.625< Dietary Na/K ratio<6.053)	149	84 (56.4)		
Dinner energy ratio>39.1% (Dietary Na/K ratio>6.053)^b^	150	86 (57.3)		

Na/K ratio, sodium-to-potassium ratio.

^a^The group with the lowest prevalence of hypertension.

^b^The group with the highest prevalence of hypertension.

^c^Hypertension was defined as average systolic blood pressure ⩾ 140 mm Hg and/or average diastolic blood pressure ⩾ 90 mm Hg or currently receiving hypertension treatments.

**Table 4.** Logistic regression for interaction effect of dinner energy ratio and dietary Na/K ratio on hypertension^b^

Dietary Na/K ratio	*n*	Dinner energy ratio			*P*-value

		T1 (<30.2%)	T2 (30.2–39.1%)	T3 (>39.1%)	
Unadjusted OR (95% CI)	1,731				0.003
Q1 (<2.348)		1.490 (0.956–2.321)	1.000 (reference)	1.220 (0.756–1.969)	
Q2 (2.348–3.625)		1.363 (0.841–2.207)	1.189 (0.763–1.853)	1.454 (0.935–2.263)	
Q3 (3.625–6.053)		1.914 (1.222–2.999)	1.211 (0.758–1.933)	2.133 (1.360–3.347)	
Q4 (>6.053)		2.105 (1.350–3.282)	1.707 (1.061–2.746)	2.218 (1.414–3.479)	
Adjusted OR (95% CI)^a^	1,504				0.001
Q1 (<2.348)		1.549 (0.933–2.572)	1.000 (reference)	1.545 (0.885–2.697)	
Q2 (2.348–3.625)		1.568 (0.874–2.814)	1.380 (0.834–2.283)	2.166 (1.306–3.593)	
Q3 (3.625–6.053)		2.049 (1.202–3.493)	1.093 (0.630–1.898)	2.984 (1.758–5.066)	
Q4 (>6.053)		2.061 (1.195–3.553)	2.039 (1.152–3.609)	2.631 (1.552–4.459)	

CI, confidence interval; Na/K ratio, sodium-to-potassium ratio; OR, odds ratio; Q, quartile; T, tertile.

^a^Adjusted for age, sex, marital status, occupation, body mass index, physical activity, excessive drinking, family history of hypertension, cardiovascular disease, chronic urinary system disease.

^b^Hypertension was defined as average systolic blood pressure ⩾ 140 mm Hg and/or average diastolic blood pressure ⩾ 90 mm Hg or currently receiving hypertension treatments.

### Simple effects of dinner energy ratio and dietary Na/K ratio on hypertension

In all the dietary Na/K ratio groups (Q1, Q2, Q3, and Q4), the ORs of hypertension prevalence in the group with dinner energy ratio less than 30.2% (T1) and dinner energy ratio greater than 39.1% (T3) were higher than the group with dinner energy ratio between 30.2% and 39.1% (T2). Among participants, those with a dietary Na/K ratio greater than 6.053 (Q4), participants with a dinner energy ratio less than 30.2% (T1), between 30.2–39.1% (T2), or greater than 39.1% (T3) had a high prevalence of hypertension, with ORs of 2.061 (95% CI, 1.195–3.553), 2.039 (95% CI, 1.152–3.609) and 2.631 (95% CI, 1.552–4.459) respectively, compared with participants with a dinner energy ratio of 30.2–39.1% and a dietary Na/K ratio less than 2.348. Thus, a low or a high dinner energy ratio enhanced the effect of dietary Na/K ratio on hypertension (Table 4).

As the dietary Na/K ratio increased, the ORs of hypertension prevalence almost always increased in all the dinner energy ratio groups (T1, T2, and T3). Among participants with a dinner energy ratio greater than 39.1% (T3), participants with a dietary Na/K ratio less than 2.348 (Q1), from 2.348–3.625 (Q2), from 3.625–6.053 (Q3), or greater than 6.053 (Q4) had a high prevalence of hypertension, with ORs of 1.545 (95% CI, 0.885–2.697), 2.166 (95% CI, 1.306–3.593), 2.984 (95% CI, 1.758–5.066) and 2.631 (95% CI, 1.552–4.459) respectively, compared with participants who with a dinner energy ratio of 30.2–39.1% and a dietary Na/K ratio less than 2.348. Thus, a high dietary Na/K ratio enhanced the effect of dinner energy ratio on hypertension (Table 4).

### Sensitivity analyses

The interaction between dinner energy ratio and dietary Na/K ratio on hypertension still existed after excluding patients with tumors, cardiovascular disease, and chronic urinary system disease (eTable 3). Compared with participants with a dinner energy ratio of 30.2–39.1% (T2), a high or a low dinner energy ratios were still independently related to hypertension after excluding patients with chronic urinary system disease (T1 vs T2: adjusted OR 1.346; 95% CI, 1.002–1.808; T3 vs T2: adjusted OR 1.671; 95% CI, 1.245–2.241) (eTable 4). The results of sensitivity analysis also showed that, compared with participants whose dinner energy ratio was 30.2–39.1% (T2), a low dinner energy ratio (<30.2%) was not related to hypertension (adjusted OR 1.330; 95% CI, 0.993–1.782) after excluding patients with tumors (eTable 5); the result was similar after excluding patients with cardiovascular disease (adjusted OR 1.285; 95% CI, 0.958–1.724) (eTable 6).

## DISCUSSION

In our study, the prevalence of hypertension was 49.1% among adults in Inner Mongolia, which was higher than the prevalence among Chinese adults aged ≥18 years (27.90%).^37^ Our results showed that higher dietary sodium intake and lower dietary potassium intake were both related to high prevalence hypertension. Our participants with an average dietary sodium intake of 5.1 g/day, higher than 5.0 g/day in adults in China National Nutrition and Health Surveillance from 2010 to 2012,^38^ and higher than 4.1 g/day in adults in a cohort study from nine provinces of China in 2015.^39^ However, the average daily dietary potassium intake of 1.2 g was lower than that in the cohort study (1.5 g/day).^39^ The diet of Inner Mongolians was characterized by less intake of fresh fruit and vegetables and more intake of oily and salty foods, which might be contribute to the high sodium intake of our participants and low potassium intake.^30^ Previous study had shown that high sodium intake was one of the top three dietary risk factors worldwide,^40^ which was an independent risk factor for hypertension. Conversely, sodium reduction could lower blood pressure in people who were hypertensive or normotensive.^41^ High dietary sodium intake was also associated with arterial stiffness,^42^ an expansion in circulatory volume, an increase in flow, and increased blood pressure.^43^ Previous evidence had also shown that low dietary potassium intake was associated with the incident of hypertension.^44^ Conversely, increasing potassium supplementation could lower blood pressure significantly,^45^ which might because of increased plasma potassium inducing endothelium dependent vasodilation,^46^ decreasing the sensitivity to catecholamine-associated vasoconstriction, reducing vascular oxidative stress and inflammation, and promoting sodium excretion.^47^^,^^48^

However, the effect of potassium on blood pressure is contingent on the concurrent intake of sodium.^49^ Our study confirmed that a high dietary Na/K ratio was independently associated with a high prevalence of hypertension, and the average dietary Na/K ratio was 5.0. A Korean multi-rural communities cohort study showed that the average dietary Na/K ratio was 1.47 in adults aged 40 years and older, and a dietary Na/K ratio greater than 1.0 was related to subclinical vascular damage.^50^ Similar to our results, data from the longitudinal China Health and Nutrition Survey also showed that a high dietary Na/K ratio was associated with a high risk of increased diastolic blood pressure in North China.^51^ Another study showed that a low potassium diet could cause sodium sensitivity with a significant dose dependence; conversely, a potassium-rich diet could counteract sodium sensitivity, whether it is in people with normal blood pressure or hypertension.^52^ A survey from three main linguistic regions in Switzerland showed that hypertensive people had a higher 24-hour urinary Na/K ratio than normotensive people.^53^ Another cross-sectional study from the United States showed that dietary excessive Na intake and K deficiency were closely related to the pathogenesis of hypertension.^54^ A recent study in a general Japanese population found that a urinary Na/K ratio greater than 5.2 was significantly associated with increased prevalence of hypertension.^55^

The present study showed that dinner energy ratio was also independently related to the prevalence of hypertension among adults in Inner Mongolia, and the average dinner energy ratio was 35.0%, which was higher than that observed in previous studies. The average dinner energy ratio was 34.0% for Tianjin residents^56^ and 27.0% for Chinese Children in a longitudinal analysis.^57^ Compared with people whose dinner energy ratio was 30.2–39.1% (T2), a high dinner energy ratio (>39.1%) was related to an increasing prevalence of hypertension (adjusted OR 1.716; 95% CI, 1.302–2.261). A study on middle-aged adults showed that dinner energy ratio was not related to high blood pressure,^18^ which was inconsistent with our results. This might be because their participants who had high dinner energy ratio always had low sodium intake, whereas the average daily sodium intake of our high dinner energy ratio population was high (5.2 g). A recent study from the China Health and Nutrition Survey showed that Chinese adults whose proportion of dinner energy was over 40% had higher dyslipidemia risk,^58^ and a multi-center randomized trial of the Anglo-Scandinavian Cardiac Outcomes Trial (ASCOT) Legacy Study from the United Kingdom showed that dyslipidemia was associated with high blood pressure.^59^ When dinner energy ratio was higher than 40% over years, it was associated with long-term hyperglycemia risk.^60^ However, hyperglycemia might exert many cellular effects and lead to endothelial dysfunction and vascular inflammation,^61^ both of which were risk factors for hypertension. The above studies all supported our results.

Interestingly, we found a U-shaped relationship between dinner energy ratio and hypertension: compared with people whose dinner energy ratio was 30.2–39.1% (T2), a higher dinner energy ratio (>39.1%) was related to an increased prevalence of hypertension, and a lower dinner energy ratio (<30.2%) was also related to an increasing prevalence of hypertension (adjusted OR 1.371; 95% CI, 1.036–1.814). In China, the appropriate dinner energy ratio was considered to be 30–40%,^18^ which was not evidence-based. Our study suggested that dinner energy ratio of 30.2–39.1% was relatively healthy. Low dietary energy intake may be due to the reduced secretion of ghrelin.^62^ Ghrelin could enhance vascular activity and angiogenesis and regulate blood pressure.^63^ A study on young Japanese women showed that low energy intake might result in low sleep quality,^64^ and low sleep quality was related to hypertension.^65^ A meta-analysis showed that there was no significant difference in weight change between a large and a small dinner,^66^ which indirectly supported our results. Our further sensitivity analysis showed that dinner energy ratio less than 30.2% (T1) was not related to hypertension after separately excluding participants with tumor or cardiovascular disease. Older participants, including those people who have tumor or cardiovascular disease, might change their dietary energy intake, and there might be reverse causality in our cross-sectional study among these people. The National Nutrition and Health Surveillance conducted from 2010–2012 across China showed that people aged 60 years and older had a high percentage of skipping dinner.^67^ Our participants whose dinner energy ratio less than 30.2% (T1) were older than participants with a dinner energy ratio of 30.2–39.1% (T2), and they had a higher dietary Na/K ratio, lower percentage of married status, and more tumor or cardiovascular disease. Similarly, a prospective Chinese breast cancer cohort study showed that breast cancer patients reported a significant decrease in energy intake.^68^

In our study, there was a significant interaction between dietary Na/K ratio and dinner energy ratio on hypertension among adults in Inner Mongolia. Participants with a high dinner energy ratio and high dietary Na/K ratio had higher ORs of hypertension, compared with those people whose dinner energy ratio was 30.2–39.1% and combined dietary Na/K ratio less than 2.348. Similar to our results, the frequency of high energy-dense and salty food intake has previously been found to be associated with diastolic hypertension in Spanish children.^69^ Another previous study^70^ found that chronic excessive sodium intake sends too many abnormal signals to the brain’s feeding center, causing people to eat too much, which led to excessive energy intake and an increased risk of hypertension. Excessive sodium intake might cause inflammatory damage to the vascular endothelium,^71^ and excessive energy intake might also cause an inflammatory reaction of the blood vessels,^72^ leading to vascular endothelial dysfunction and thus to increased blood pressure. Moreover, excessive energy intake might lead to insulin resistance, which increased the reabsorption of sodium ions into the renal tubules, leading to water and sodium retention in the body and causing high blood pressure.^73^^,^^74^ However, the concomitant intake of sodium was also related to the effect of potassium on blood pressure.^75^ Low potassium might reduce the production of nitric oxide in endothelial cells and increase the release of growth factor-β, stiffening the vascular walls,^76^ which might result in high blood pressure.

We also found that participants whose dinner energy ratio was less than 30.2% and their dietary Na/K ratio was high had higher ORs of hypertension than those people whose dinner energy ratio was 30.2–39.1% and combined dietary Na/K ratio was less than 2.348. In China, residents always had high dietary sodium intake and low potassium intake,^39^ and another study showed that the blood pressure was elevated with increasing sodium intake even at lower energy intake,^27^ which could support our results. We also found that, at the same level of dinner energy ratio, a high dietary Na/K ratio was related to an increased OR of hypertension across all dinner energy ratio groups. This suggested that the dinner energy ratio was an important influential factor for the association of dietary Na/K ratio with the prevalence of hypertension.

Inner Mongolia has relatively developed animal husbandry and is rich in horses, cattle, sheep, pigs, and other livestock. Lots of residents in Inner Mongolia are more likely to consume too much high-fat food, such as a big piece of pork, beef, or mutton, and the intake of high-fat food was more often in dinner than breakfast. They also have a habit of drinking excessively with high-fat food at dinner. Moreover, many residents of Inner Mongolia had a dietary habit of high sodium intake. With the integration of Mongolian and Han culture, soy sauce food had become an indispensable non-staple food for residents.^77^ The daily intake frequency of pickles, salted beef jerky, and cured meat was high, and low potassium diets with inadequate intake of fruits and vegetables, legumes, and nuts are problematic in residents who lived in Inner Mongolia.^23^^,^^78^ All these above factors could lead to a high dietary Na/K ratio. Our results indicated that the consistent presence of both high dietary Na/K ratio intake and inadequate dinner energy ratio was related to a high prevalence of hypertension.

To the best of our knowledge, this study is the first exploration to assess the interaction of dinner energy ratio and dietary Na/K ratio on hypertension. From the perspective of the population of Inner Mongolia, our results provide a basis for future dietary behavior modification with respect to dietary Na/K ratio and dinner energy ratio. Our study data were from a surveillance survey of chronic disease and nutrition among adults in Inner Mongolia, and the data are representative of the population in that region. In the data analysis, we also adjusted for age, sex, marital status, occupation, BMI, physical activity, excessive drinking, family history of hypertension, cardiovascular disease, and chronic urinary system disease to reduce confounding bias. However, our study was a cross-sectional, so despite effective quality control and adjustment for confounding factors, a certain amount of bias is unavoidable. Since 24-hour urine collection was considered the “gold standard” method to assess daily intake of both Na and K in populations, while the method of our study was Na and K in foods and condiments, this could cause some bias. Although the study findings demonstrated the interaction of dinner energy ratio and dietary Na/K ratio on hypertension, long-term follow-up studies should be conducted to determine whether there is a causal relationship between these factors and hypertension.

### Conclusions

The results of this study indicated that both inadequate dinner energy ratio and high dietary Na/K ratio were highly prevalent among adults in Inner Mongolia. Our study revealed that both a high or a low dinner energy ratio and a high dietary Na/K ratio were independently related to hypertension. Crucially, the dinner energy ratio was found to interact significantly with dietary Na/K ratio in the prevalence of hypertension among adults in Inner Mongolia. We advocated a balanced diet (dinner energy ratio not small nor large) and a low dietary Na/K ratio for reducing the prevalence of hypertension.

## SUPPLEMENTARY MATERIAL

eTable 1. The characteristics of the subjects in each of the tertile of the dinner energy ratio (n = 1,731)

eTable 2. Association between dietary sodium or potassium intake and prevalent hypertension

eTable 3. Sensitivity analysis of the interaction effects of dinner energy ratio and dietary Na/K ratio on hypertension ^a^ after excluding patients with tumor, cardiovascular disease, and chronic urinary system disease

eTable 4. Sensitivity analysis of the odds ratios of hypertension^a^ for dinner energy ratio and dietary Na/K ratio after excluding patients with chronic urinary system disease

eTable 5. Sensitivity analysis of the adjusted ^c^ odds ratios of hypertension^a^ for dinner energy ratio and dietary Na/K ratio after excluding patients with tumor

eTable 6. Sensitivity analysis of the odds ratios of hypertension^a^ for dinner energy ratio and dietary Na/K ratio after excluding patients with cardiovascular disease
