# Supplementary material for: Interaction of Dietary Sodium-to-potassium Ratio and Dinner Energy Ratio on Prevalence of Hypertension in Inner Mongolia, China
Source: J Epidemiol. 2023 Nov 5;33(11):547–55. doi: 10.2188/jea.JE20220045 (PMC10518379; doi:10.2188/jea.JE20220045)
Supplement: Supplementary file 1 [file je-33-547-s001.pdf]

eTable 1. The characteristics of the subjects in each of the tertile of the dinner energy ratio (n=1,731)

**Variables**	**Dinner energy ratio**			***F*/χ^2**	***P*-value**
	T1, n (%) 577 (33.3)	T2, n (%) 577 (33.3)	T3, n (%) 577 (33.3)		
Age, years, mean (SD)	53.9 (13.1)	51.9 (12.5)	51.0 (11.7)	9.647	<0.001[a]
Sex					
Men	266 (46.1)	287 (49.7)	278 (48.2)	1.541	0.463
Women	311 (53.9)	290 (50.3)	299 (51.8)		
Ethnicity					
Han	482 (83.5)	474 (82.1)	472 (81.8)	2.388	0.665
Mongolian	72 (12.5)	85 (14.7)	80 (13.9)		
Other ethnic minority	23 (4.0)	18 (3.1)	25 (4.3)		
Marital status					
Married	515 (89.6)	544 (94.6)	547 (95.0)	19.731	0.001[a]
Unmarried	18 (3.1)	15 (2.6)	12 (2.1)		
Other marital status	42 (7.3)	16 (2.8)	17 (3.0)		
Occupation					
Agriculture and farming	216 (37.4)	223 (38.6)	281 (48.7)	32.857	0.001[a]
Production equipment operation and service	59 (10.2)	67 (11.6)	46 (8.0)		
Enterprises and public institutions	34 (5.9)	27 (4.7)	28 (4.9)		
Unemployed	22 (3.8)	14 (2.4)	26 (4.5)		
Homemaker	109 (18.9)	99 (17.2)	86 (14.9)		
Retired	59 (10.2)	57 (9.9)	31 (5.4)		
Other occupation	78 (13.5)	90 (15.6)	79 (13.7)		
Family history of hypertension					
Yes	214 (37.1)	238 (41.2)	223 (38.6)	2.142	0.343
No	363 (62.9)	339 (58.8)	354 (61.4)		
Dietary sodium, g, mean (SD)	5.1 (4.4)	5.0 (4.1)	5.2 (4.1)	0.166	0.847
Dietary potassium, g, mean (SD)	1.3 (0.6)	1.4 (0.6)	1.3 (0.7)	10.944	<0.001[a]
Dietary Na/K ratio	4.9 (4.4)	4.3 (4.0)	5.0 (4.2)	3.747	0.024 [a]
Excessive drinking					
No	534 (92.5)	522 (90.5)	499 (86.5)	12.005	0.002[a]
Yes	43 (7.5)	55 (9.5)	78 (13.5)		
Smoking					

Non-current smoker	424 (73.5)	385 (66.7)	370 (64.1)	12.400	0.002 [a]
Current smoker	153 (26.5)	192 (33.3)	207 (35.9)		
BMI					
Underweight/normal	206 (36.9)	219 (38.0)	254 (45.3)	9.102	0.011[a]
Overweight	240 (42.9)	247 (42.9)	214 (38.1)		
Obese	113 (20.2)	110 (19.1)	93 (16.6)		
Physical activity, METs-min/w, mean (SD)	5,742.4 (4,443.4)	5,946.9 (5,127.4)	5,962.7 (5,636.7)	0.293	0.746
Tumor					
No	537 (93.2)	547 (94.8)	552 (95.7)	3.427	0.180
Yes	39 (6.8)	30 (5.2)	25 (4.3)		
Cardiovascular disease					
No	518 (89.9)	520 (90.1)	522 (90.5)	0.097	0.953
Yes	58 (10.1)	57 (9.9)	55 (9.5)		
Chronic urinary system disease					
No	532 (92.4)	516 (89.4)	507 (87.9)	6.598	0.037[a]
Yes	44 (7.6)	61 (10.6)	70 (12.1)		

BMI, body mass index; METs-min/w, metabolic equivalents of task-minutes/week; Na/K ratio, sodium-to-potassium ratio; SD, standard deviation; T, tertile.

[a] $P<0.05$.

eTable 2. Association between dietary sodium or potassium intake and prevalent hypertension

Variables	Unadjusted (n=1,860)		*P*-value	Adjusted [a] (n=1,626)		*P*-value
	OR	95% CI		OR	95% CI	
Dietary sodium, g	1.017	(0.995–1.041)	0.138	1.030	(1.003–1.058)	0.029 [b]
Dietary potassium, g	0.693	(0.600–0.801)	<0.001 [b]	0.697	(0.583–0.832)	<0.001 [b]

CI, confidence interval; OR, odds ratio.

[a] Adjusted for age, sex, marital status, occupation, body mass index, physical activity, excessive drinking, family history of hypertension, cardiovascular disease, chronic urinary system disease.

[b] $P<0.05$.

[c] Hypertension was defined as average systolic blood pressure ≥140 mm Hg and/or average diastolic blood pressure ≥90 mm Hg or currently receiving hypertension treatments.

eTable 3. Sensitivity analysis of the interaction effects of dinner energy ratio and dietary Na/K ratio on hypertension [a] after excluding patients with tumor, cardiovascular disease, and chronic urinary system disease

**Dietary Na/K ratio**	**Dinner energy ratio**		
	T1 (<30.2%)	T2 (30.2–39.1%)	T3 (>39.1%)
Adjusted OR (95% CI) [b]			
Q1(<2.348)	1.549 (0.933–2.572)	1.000 (reference)	1.545 (0.885–2.697)
Q2(2.348–3.625)	1.568 (0.874–2.814)	1.380 (0.834–2.283)	2.166 (1.306–3.593)
Q3(3.625–6.053)	2.049 (1.202–3.493)	1.093 (0.630–1.898)	2.984 (1.758–5.066)
Q4(>6.053)	2.061 (1.195–3.553)	2.039 (1.152–3.609)	2.631 (1.552–4.459)
Adjusted OR (95% CI) [c]			
Q1(<2.348)	1.677 (0.975–2.884)	1.000 (reference)	1.552 (0.855–2.815)
Q2(2.348–3.625)	1.462 (0.781–2.735)	1.576 (0.919–2.704)	2.597 (1.505–4.481)
Q3(3.625–6.053)	2.384 (1.351–4.204)	1.170 (0.650–2.106)	3.248 (1.831–5.716)
Q4(>6.053)	2.142 (1.202–3.818)	2.403 (1.296–4.457)	2.491 (1.414–4.387)
Adjusted OR (95% CI) [d]			
Q1(<2.348)	1.537 (0.901–2.621)	1.000 (reference)	1.455 (0.812–2.608)
Q2(2.348–3.625)	1.645 (0.893–3.031)	1.350 (0.802–2.274)	2.081 (1.226–3.534)
Q3(3.625–6.053)	1.954 (1.121–3.408)	1.160 (0.653–2.060)	2.823 (1.628–4.897)
Q4(>6.053)	1.849 (1.043–3.277)	2.018 (1.112–3.664)	2.367 (1.360–4.122)
Adjusted OR (95% CI) [e]			
Q1(<2.348)	1.308 (0.769–2.223)	1.000 (reference)	1.479 (0.830–2.635)
Q2(2.348–3.625)	1.409 (0.761–2.610)	1.337 (0.793–2.253)	1.925 (1.139–3.253)
Q3(3.625–6.053)	2.038 (1.168–3.556)	0.980 (0.548–1.753)	2.606 (1.495–4.541)
Q4(>6.053)	1.779 (1.008–3.139)	2.039 (1.114–3.730)	2.343 (1.341–4.095)

CI, confidence interval; Na/K ratio, sodium-to-potassium ratio; OR, odds ratio; Q, quartile; T, tertile.

[a] Hypertension was defined as average systolic blood pressure ≥140 mm Hg and/or average diastolic blood pressure ≥90 mm Hg or currently receiving hypertension treatments.

[b] Patients with tumor, cardiovascular disease and chronic urinary system disease were not excluded (n=1,504), and we adjusted for age, sex, marital status, occupation, body mass index, physical activity, excessive drinking, family history of hypertension, cardiovascular disease, chronic urinary system disease.

[c] We excluded chronic urinary system disease patients (n=1,345), and we adjusted for age, sex, marital status, occupation, body mass index, physical activity, excessive drinking, family history of hypertension, cardiovascular disease.

[d] We excluded tumor patients (n=1,412), and we adjusted for age, sex, marital status, occupation, body mass index, physical activity, excessive drinking, family history of hypertension, cardiovascular disease, chronic urinary system disease.

[e] We excluded cardiovascular disease patients (n=1,368), and we adjusted for age, sex, marital status, occupation, body mass index, physical activity, excessive drinking, family history of hypertension, chronic urinary system disease.

eTable 4. Sensitivity analysis of the odds ratios of hypertension[a] for dinner energy ratio and dietary Na/K ratio after excluding patients with chronic urinary system disease

Variables	Non-exclusive (n=1,504)		Exclusive-chronic urinary system disease (n=1,345)	
	**OR (95% CI)** [c]	***P*-value**	**OR (95% CI)** [d]	***P*-value**
**Dinner energy ratio**				
T1 (<30.2%)	1.371 (1.036–1.814)	0.027 [b]	1.346 (1.002–1.808)	0.049 [b]
T2 (30.2–39.1%)	1.000 (reference)		1.000 (reference)	
T3 (>39.1%)	1.716 (1.302–2.261)	<0.001[b]	1.671 (1.245–2.241)	0.001 [b]
**Dietary Na/K ratio**				
Q1 (<2.348)	1.000 (reference)		1.000 (reference)	
Q2 (2.348–3.625)	1.255 (0.919–1.714)	0.153	1.350 (0.970–1.878)	0.075
Q3 (3.625–6.053)	1.409 (1.027–1.934)	0.034 [b]	1.510 (1.080–2.109)	0.016 [b]
Q4 (>6.053)	1.624 (1.174–2.247)	0.003 [b]	1.633 (1.159–2.301)	0.005 [b]
**Dinner energy ratio×Dietary Na/K ratio**	1.119 (1.040–1.203)	0.002 [b]	1.120 (1.035–1.212)	0.005 [b]

CI, confidence interval; Na/K ratio, sodium-to-potassium ratio; OR, odds ratio; Q, quartile; T, tertile.

[a] Hypertension was defined as average systolic blood pressure ≥140 mm Hg and/or average diastolic blood pressure ≥90 mm Hg or currently receiving hypertension treatments.

[b] $P<0.05$.

[c] Adjusted for age, sex, marital status, occupation, body mass index, physical activity, excessive drinking, family history of hypertension, cardiovascular disease, chronic urinary system disease.

[d] Adjusted for age, sex, marital status, occupation, body mass index, physical activity, excessive drinking, family history of hypertension, cardiovascular disease.

eTable 5. Sensitivity analysis of the adjusted [c] odds ratios of hypertension[a] for dinner energy ratio and dietary Na/K ratio after excluding patients with tumor

Variables	Non-exclusive (n=1,504)		Excluded-tumor (n=1,412)	
	OR (95% CI) [c]	*P*-value	OR (95% CI) [d]	*P*-value
**Dinner energy ratio**				
T1 (<30.2%)	1.371 (1.036–1.814)	0.027 [b]	1.330 (0.993–1.782)	0.056
T2 (30.2–39.1%)	1.000 (reference)		1.000 (reference)	
T3 (>39.1%)	1.716 (1.302–2.261)	<0.001[b]	1.624 (1.219–2.165)	0.001 [b]
**Dietary Na/K ratio**				
Q1 (<2.348)	1.000 (reference)		1.000 (reference)	
Q2 (2.348–3.625)	1.255 (0.919–1.714)	0.153	1.247 (0.900–1.729)	0.184
Q3 (3.625–6.053)	1.409 (1.027–1.934)	0.034 [b]	1.411 (1.011–1.971)	0.043 [b]
Q4 (>6.053)	1.624 (1.174–2.247)	0.003 [b]	1.537 (1.084–2.180)	0.016 [b]
**Dinner energy ratio×Dietary Na/K ratio**	1.119 (1.040–1.203)	0.002 [b]	1.120 (1.035–1.211)	0.005 [b]

CI, confidence interval; Na/K ratio, sodium-to-potassium ratio; OR, odds ratio; Q, quartile; T, tertile.

[a] Hypertension was defined as average systolic blood pressure ≥140 mm Hg and/or average diastolic blood pressure ≥90 mm Hg or currently receiving hypertension treatments.

[b] $P<0.05$.

[c] Adjusted for age, sex, marital status, occupation, body mass index, physical activity, excessive drinking, family history of hypertension, cardiovascular disease, chronic urinary system disease.

[d] Adjusted for age, sex, marital status, occupation, body mass index, physical activity, excessive drinking, family history of hypertension, cardiovascular disease, chronic urinary system disease.

eTable 6. Sensitivity analysis of the odds ratios of hypertension[a] for dinner energy ratio and dietary Na/K ratio after excluding patients with cardiovascular disease

**Variables**	**Non-exclusive (n=1,504)**		**Excluded-cardiovascular disease (n=1,368)**	
	**OR (95% CI)** [c]	***P*-value**	**OR (95% CI)** [d]	***P*-value**
**Dinner energy ratio**				
T1 (<30.2%)	1.371 (1.036–1.814)	0.027 [b]	1.285 (0.958–1.724)	0.094
T2 (30.2–39.1%)	1.000 (reference)		1.000 (reference)	
T3 (>39.1%)	1.716 (1.302–2.261)	<0.001[b]	1.588 (1.190–2.120)	0.002 [b]
**Dietary Na/K ratio**				
Q1 (<2.348)	1.000 (reference)		1.000 (reference)	
Q2 (2.348–3.625)	1.255 (0.919–1.714)	0.153	1.244 (0.899–1.722)	0.187
Q3 (3.625–6.053)	1.409 (1.027–1.934)	0.034 [b]	1.414 (1.015–1.970)	0.041 [b]
Q4 (>6.053)	1.624 (1.174–2.247)	0.003 [b]	1.608 (1.143–2.263)	0.006 [b]
**Dinner energy ratio×Dietary Na/K ratio**	1.119 (1.040–1.203)	0.002 [b]	1.134 (1.049–1.225)	0.002 [b]

CI, confidence interval; Na/K ratio, sodium-to-potassium ratio; OR, odds ratio; Q, quartile; T, tertile.

[a] Hypertension was defined as average systolic blood pressure ≥140 mm Hg and/or average diastolic blood pressure ≥90 mm Hg or currently receiving hypertension treatments.

[b] $P<0.05$.

[c] Adjusted for age, sex, marital status, occupation, body mass index, physical activity, excessive drinking, family history of hypertension, cardiovascular disease, chronic urinary system disease.

[d] Adjusted for age, sex, marital status, occupation, body mass index, physical activity, excessive drinking, family history of hypertension, chronic urinary system disease.
